# Delayed Gastric Emptying After Pancreaticoduodenectomy: Impact of Reconstruction Techniques

**DOI:** 10.7759/cureus.99342

**Published:** 2025-12-15

**Authors:** Sergio Isidro Gamboa-Hoil

**Affiliations:** 1 Surgical Oncology, Mexican Social Security Institute, Mérida, MEX

**Keywords:** billroth ii, delayed gastric emptying, duodenojejunostomy, gastrojejunostomy, pancreaticoduodenectomy, pancreatic surgery, pylorus-preserving pancreaticoduodenectomy, reconstruction techniques, roux-en-y reconstruction, whipple procedure

## Abstract

Delayed gastric emptying (DGE) is a common complication after pancreaticoduodenectomy (PD) and contributes to prolonged recovery and delayed initiation of adjuvant therapy. It is clinically significant, as it can impair oral intake and extend hospitalization. Its multifactorial pathophysiology includes mechanical, hormonal, neural, and inflammatory factors, and increasing attention has been directed toward the role of reconstruction technique. A narrative review was conducted to evaluate reconstruction strategies after PD. A non-systematic search of major databases identified studies comparing various approaches, including jejunal loop routing, anastomotic configuration, and pyloric preservation, and their impact on DGE. The available evidence remains highly variable, drawing from retrospective series, randomized trials, and prior meta-analyses. While some reconstruction approaches have been associated with lower rates of DGE in certain settings, reported outcomes are inconsistent and often limited by single-center designs or small sample sizes. Overall, comparative studies frequently fail to demonstrate a clear advantage of one reconstruction strategy over another with respect to DGE incidence. Although specific reconstruction techniques may influence its occurrence after PD, no single approach has shown consistent superiority. Variability in surgical expertise, anatomical configuration, perioperative management, and study methodology contributes to these inconsistent findings. High-quality prospective, multicenter randomized studies are needed to clarify the true impact of reconstruction technique and guide standardized surgical decision-making.

## Introduction and background

Delayed gastric emptying (DGE) after pancreaticoduodenectomy (PD) was first described by Warshaw in 1985 [1]. According to the International Study Group of Pancreatic Surgery (ISGPS), DGE is classified into grades A, B, and C based on the duration of nasogastric tube dependence, need for tube reinsertion, and timing of solid-food tolerance. Grade A is defined as the requirement for a nasogastric tube between postoperative days (PODs) 4 and 7, or reinsertion due to nausea or vomiting after POD 3, with intolerance to solid food on POD 7 but resolution by POD 14. Grade B involves nasogastric decompression between POD 8 and 14, or reinsertion after POD 7, with inability to tolerate solid food on POD 14 but recovery by POD 21. Grade C is characterized by nasogastric tube requirement or reinsertion beyond POD 14, with persistent intolerance to solid food after POD 21 [2].

DGE is one of the most frequent complications following PD, with reported incidence varying widely across studies depending on reconstruction technique, perioperative management, and patient factors [1,3-5]. Clinically, DGE may result in prolonged hospitalization, delayed resumption of adequate oral intake, and postponement of adjuvant therapy, thereby adversely affecting oncologic timelines and increasing healthcare costs [2,6]. Its high prevalence and clinical relevance have motivated ongoing efforts to optimize reconstruction strategies to mitigate this complication.

The mechanisms contributing to DGE remain multifactorial and incompletely understood. Hormonal factors have been implicated, particularly the loss of motilin-secreting mucosa with duodenal resection, which disrupts migrating motor complexes and impairs gastric motility. Neural mechanisms, including vagal disruption, may lead to dysregulated antral and pyloric contractility [7]. Gastric dysrhythmias have also been observed during the immediate postoperative period [8].

Mechanical factors may further contribute. Angulation, torsion, or edema at the gastrojejunostomy or duodenojejunostomy can impede luminal transit, especially when anatomical tension is present [9]. Postoperative gastroparesis may exacerbate gastric distension, promoting anastomotic kinking. Additionally, minor pancreatic leaks, peripancreatic inflammation, and intra-abdominal collections have been associated with secondary impairment of gastric emptying [9,10].

Given that DGE arises from overlapping hormonal, neural, mechanical, and inflammatory mechanisms, a detailed assessment of how reconstruction techniques influence these pathways is essential to guide surgical decision-making.

## Review

Search strategy and methods

A non-systematic literature search was conducted using PubMed, MEDLINE, and Google Scholar to identify studies published between 1985 and 2025. Search terms included “pancreaticoduodenectomy”, “delayed gastric emptying”, “reconstruction technique”, “antimesenteric”, “retromesenteric”, “antecolic”, “retrocolic”, “Billroth I”, “Billroth II”, “Roux-en-Y”, “Braun enteroenterostomy”, “classic pancreaticoduodenectomy”, “pylorus-preserving pancreaticoduodenectomy”, and “pylorus-resecting pancreaticoduodenectomy”. Reference lists from key articles, systematic reviews, and meta-analyses were also reviewed to identify additional eligible studies. All study designs reporting postoperative outcomes related to reconstruction technique were considered, with inclusion limited to English-language publications.

A total of 31 studies met the inclusion criteria and were included in the narrative synthesis. Although more than 50 references were initially identified, studies were excluded if they lacked DGE-specific outcomes, did not adequately describe the reconstruction technique, or involved very small case series.

Inclusion and Exclusion Criteria

Studies were included if they (1) were published in English; (2) reported postoperative outcomes related to reconstruction techniques after PD; and (3) involved any study design, including randomized controlled trials (RCTs), prospective or retrospective cohorts, and systematic reviews or meta-analyses. Studies were excluded if they were case reports, small series with fewer than five patients, lacked DGE-specific outcomes, or consisted of narrative reviews without a focus on reconstruction approaches.

Risk of Bias Consideration

Given the narrative nature of this review, no formal risk-of-bias assessment tool was applied. Nevertheless, methodological limitations of individual studies are acknowledged and are addressed within the Discussion and Limitations sections.

Impact of reconstruction techniques on DGE

Jejunal Loop Route: Antimesenteric vs. Retromesenteric

Two retrospective studies from Korea and Canada comparing antimesenteric and retromesenteric jejunal loop ascent reported higher DGE rates with retromesenteric positioning (Park et al. [11]: 31.7% vs. 6.5%; p < 0.05; Butler et al. [12]: mean 11 vs. 7.2 days until gastric recovery; p < 0.05). Park et al. included only patients undergoing pylorus-preserving PD (PPPD), whereas the cohort evaluated by Butler et al. incorporated technical modifications that may have influenced outcomes, despite similar overall DGE incidences between the studies (24% and 28%, respectively).

**Figure 1:**
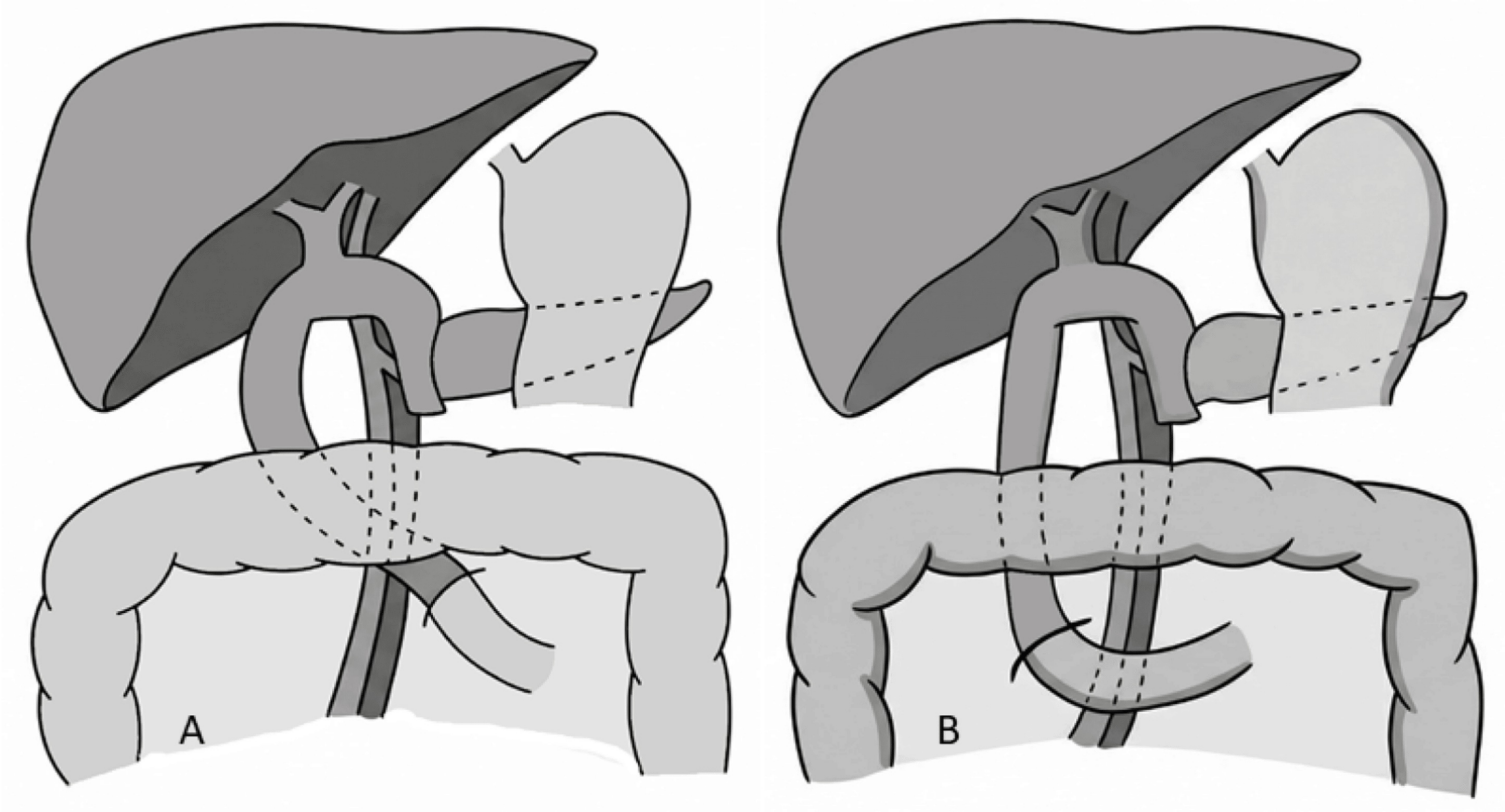
Schematic comparison of jejunal limb elevation pathways after PD (A) Retromesenteric route: jejunal limb ascending posterior to the SMA and SMV. (B) Antimesenteric route: jejunal limb ascending anterior to the SMA and SMV. Clinical relevance: The selected loop route may influence venous drainage, edema formation, and postoperative transit dynamics, thereby affecting the risk of DGE. This schematic illustration is not drawn to scale and is intended solely to represent the reconstruction pathway. DGE, delayed gastric emptying; PD, pancreaticoduodenectomy; SMA, superior mesenteric artery; SMV, superior mesenteric vein

This difference may be explained by reduced venous drainage from the jejunal branch, resulting in edema that impairs motility and delays the recovery of peristalsis, ultimately contributing to DGE (Figure 1A, 1B) [11,12].

Given the influence of jejunal loop positioning on postoperative transit dynamics, the next consideration is the route selected for gastroenteric reconstruction.

Gastroenteric Route: Antecolic vs. Retrocolic Reconstruction

RCTs in PPPD have yielded inconsistent findings. Tani et al. [13] observed a substantially higher incidence of DGE with retrocolic reconstruction (50% vs. 5%; p = 0.0014), whereas Tamandl et al. [14] and Kakaei et al. [15] did not reproduce this effect, reporting DGE rates between 27.5% and 16.6%. In RCTs including both classic PD and PPPD, no significant difference was observed between antecolic and retrocolic reconstruction (p > 0.05), with DGE rates ranging from 30.9% to 61% [16,17]. In a retrospective series of PD with antrectomy, antecolic reconstruction showed a lower DGE rate than retrocolic reconstruction (15% vs. 21%; p = 0.021) [18]. Meta-analyses and systematic reviews by Hüttner et al. [19] and Dai et al. [20] did not identify either antecolic or retrocolic gastroenterostomy as independent risk factors for DGE.

**Figure 2:**
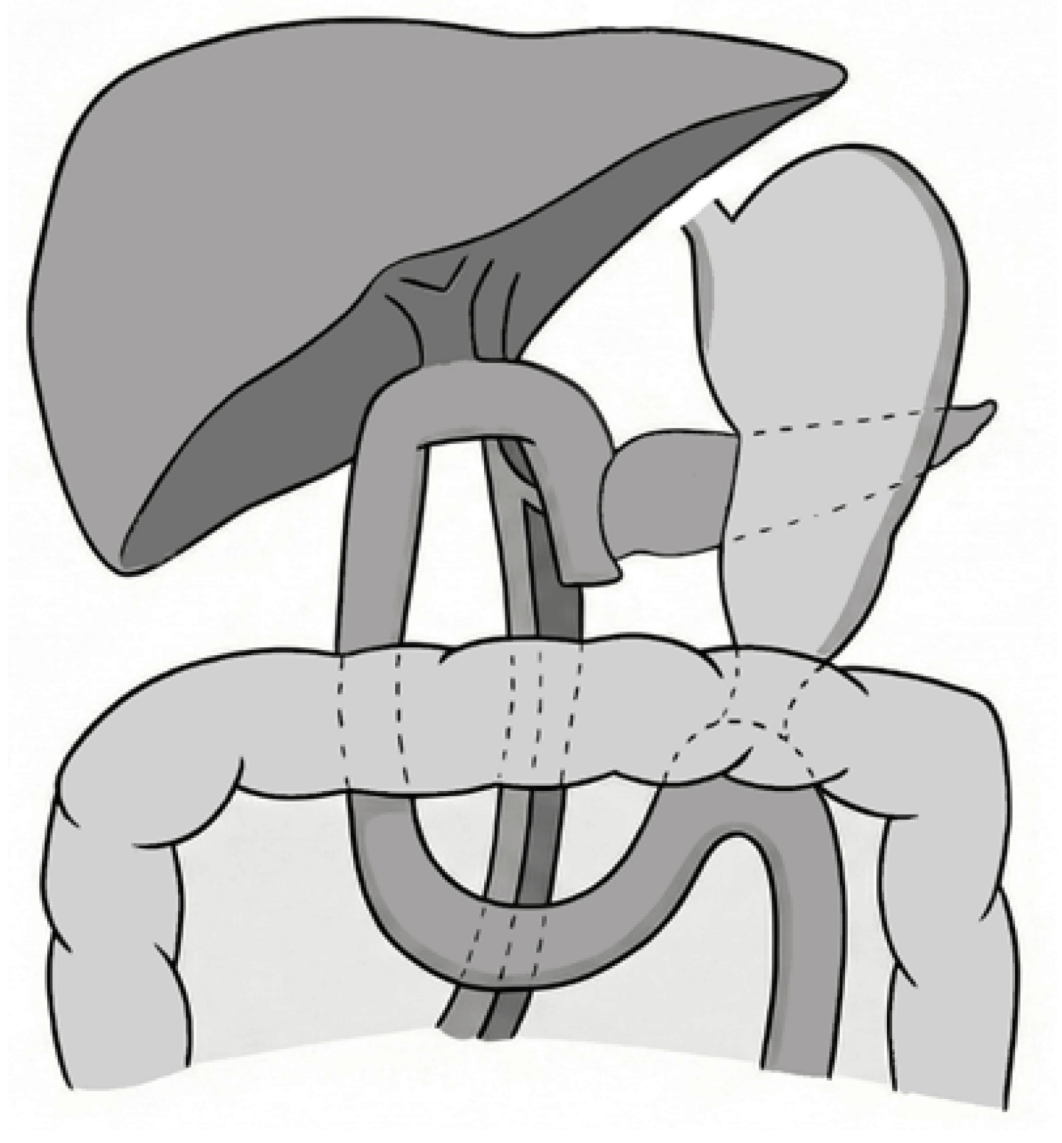
Schematic of gastroenteric limb positioning in relation to the colon after PD This figure illustrates a retrocolic anastomosis in which the jejunal limb ascends posterior to the transverse colon. Clinical relevance: The anatomical pathway of the gastroenteric limb may influence anastomotic angulation and tension, potentially affecting gastric emptying dynamics and postoperative recovery. This schematic illustration is not drawn to scale and is intended solely to represent the reconstruction pathway. PD, pancreaticoduodenectomy

Taken together, these findings indicate that although individual studies may suggest a modest benefit of the antecolic route, particularly in selected surgical contexts, the overall evidence does not demonstrate a consistent advantage of either antecolic or retrocolic reconstruction in preventing DGE (Figure 2). In summary, contemporary randomized trials and meta-analyses consistently show that both approaches are largely equivalent with respect to DGE risk.

Beyond loop orientation and route selection, the configuration of the gastroenteric anastomosis itself, such as Billroth I (BI) vs. Billroth II (BII), represents another key determinant of postoperative gastric function.

BI vs. BII Reconstruction

When comparing BI and BII reconstructions in patients undergoing PPPD, a higher incidence of DGE was observed in the BI group (Goei et al. [21]: 76% vs. 32%; p < 0.05; Kurosaki et al. [22]: BI > BII; p < 0.05). Similarly, in studies evaluating BI reconstruction in patients undergoing either classic PD or PPPD, the highest incidence of DGE was reported in the PPPD subgroup (36% vs. 4%; p not reported, Ohwada et al. [23]).

These differences may be attributable to angulation at the duodenojejunostomy site. Supporting this hypothesis, Ueno et al. [24], in a prospective non-randomized study, employed a modified BI duodenojejunostomy technique and reported no cases of DGE.

**Figure 3:**
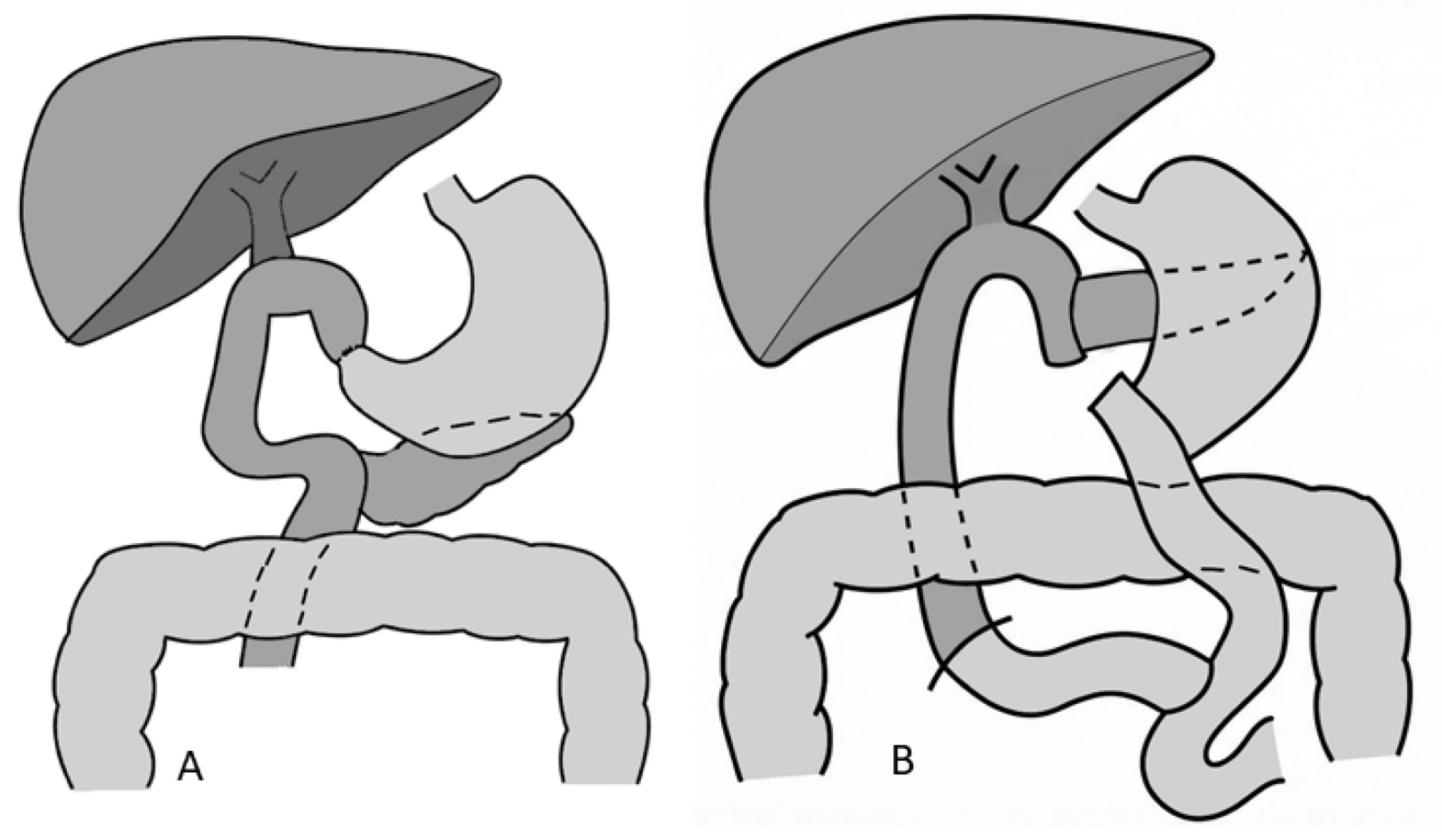
Schematic comparison of reconstructive techniques after PD (A) BI reconstruction: direct anastomosis between the gastric remnant and the jejunal limb. (B) Roux-en-Y reconstruction: jejunal limb diversion with a separate enteroenterostomy creating a Y-shaped configuration. Clinical relevance: Differences in spatial orientation and anastomotic configuration may influence gastrojejunal angulation, luminal flow, and susceptibility to DGE. This schematic illustration is not drawn to anatomical scale and is intended solely to represent the reconstruction pathway. BI, Billroth I; DGE, delayed gastric emptying; PD, pancreaticoduodenectomy

Although BI reconstruction is considered more physiological, the available space in the right upper quadrant is more limited compared with BII reconstruction, potentially contributing to technical challenges and an increased risk of DGE [21]. While BI may offer theoretical physiological advantages, BII reconstruction appears technically more favorable in the postoperative setting, and modifications of the duodenojejunostomy may help mitigate DGE risk (Figure 3A).

Building upon these configuration differences, an additional question is whether a loop or a Roux-en-Y reconstruction offers advantages in gastric emptying.

BII vs. Roux-en-Y Reconstruction

RCTs comparing BII and Roux-en-Y reconstructions have reported mixed results. Shimoda et al. [25] observed a significantly lower incidence of DGE with BII reconstruction compared with Roux-en-Y (BII 5.7% vs. Roux-en-Y 20.4%; p = 0.028), whereas Busquets et al. [26] and Herrera-Cabezón et al. [27] found no significant differences (p = 1.0 and p = 0.35, respectively).

Modifications of the Roux-en-Y technique, including isolated Roux-en-Y reconstruction, where the pancreaticojejunal loop is separated, have also been evaluated in randomized studies. However, no significant differences in DGE incidence were reported (Ke et al. [28], Tani et al. [29]; p > 0.05). Notably, these trials did not account for pylorus preservation.

A meta-analysis by Ma et al. [30] similarly found no significant differences in DGE between BII and Roux-en-Y reconstructions.

Current evidence suggests that BII and Roux-en-Y reconstructions are comparable regarding the risk of DGE, and modifications such as isolated Roux-en-Y do not appear to provide additional benefit in this context (Figure 3B). In summary, evidence from randomized studies and meta-analyses supports that BII and Roux-en-Y configurations perform similarly with respect to DGE.

Beyond the gastroenteric configuration, additional anastomotic modifications, such as Braun enteroenterostomy, have been proposed to further optimize gastric emptying.

Braun Enteroenterostomy vs. No Braun Enteroenterostomy

Retrospective studies in patients undergoing classic PD with BII reconstruction have reported conflicting results. Nikfarjam et al. [31] observed a significant reduction in DGE with Braun enteroenterostomy (p = 0.008), whereas Zhang et al. [32] and Xu et al. [33] reported no significant differences (p > 0.05). Even in prospective analyses, Wang et al. [34] did not identify a statistically significant effect (p = 0.455).

Evidence from both retrospective and randomized studies in PPPD with BII reconstruction has also been inconsistent. Watanabe et al. [35] (retrospective) identified a significant reduction in DGE (p = 0.01), Imamura et al. [36] (RCT) found no difference (p = 0.31), and Cordesmeyer et al. [37] (retrospective) observed a significant benefit (p = 0.009).

In meta-analyses, Xu et al. reported a significantly lower incidence of grade C DGE in patients receiving Braun enteroenterostomy, whereas Dai et al. did not observe this trend [20,38]. A major limitation noted by both groups was the predominantly retrospective nature of the included studies.

**Figure 4:**
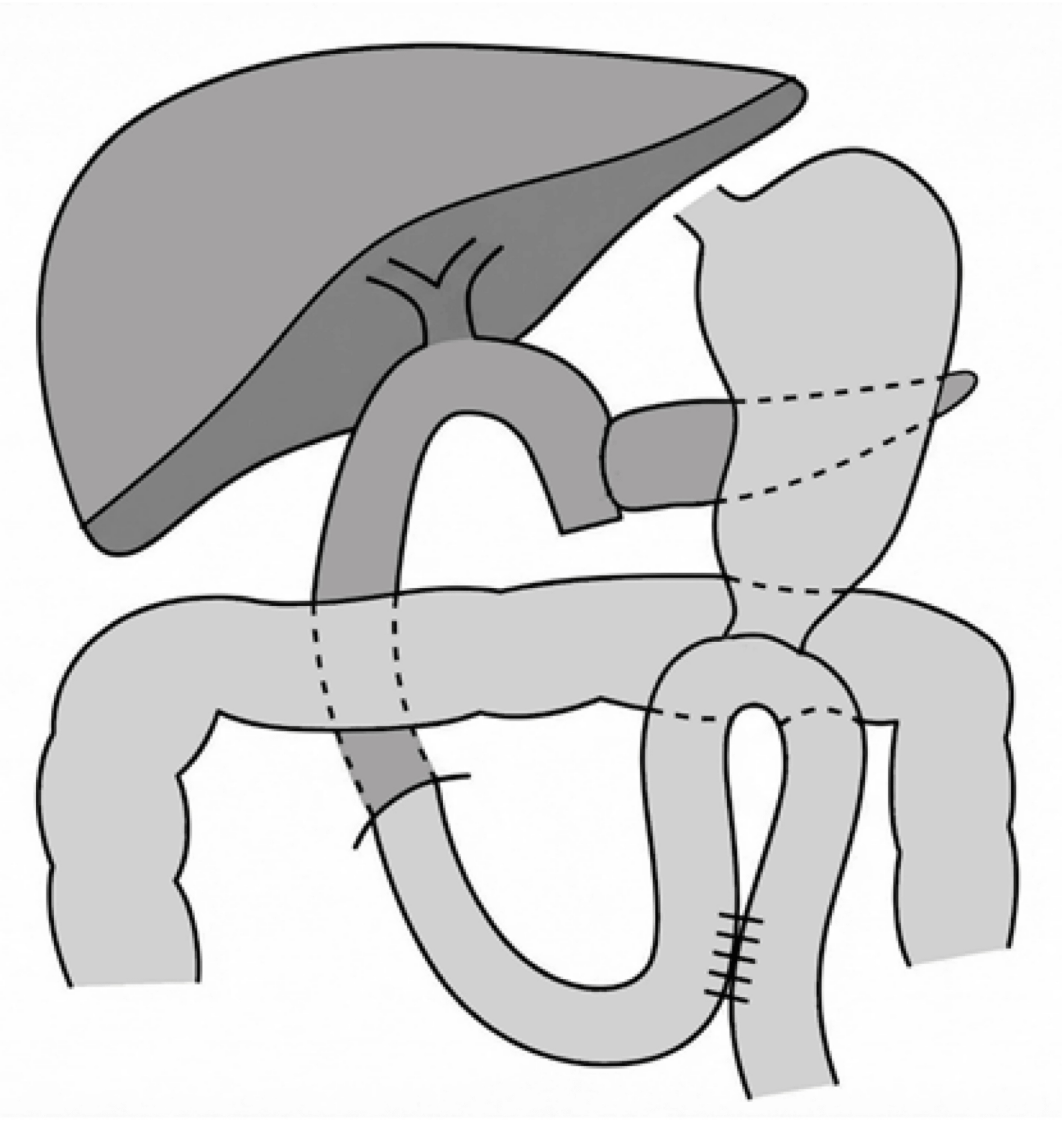
Schematic of antecolic BII reconstruction with Braun enteroenterostomy after PD The illustration depicts an antecolic gastrojejunostomy with jejunal limb elevation anterior to the mesenteric vessels, complemented by a Braun enteroenterostomy between the afferent and efferent limbs. Clinical relevance: The Braun anastomosis may reduce afferent loop stasis and facilitate gastric drainage in select settings. This schematic illustration is not drawn to anatomical scale and is intended solely to represent the reconstruction pathway. BII, Billroth II; PD, pancreaticoduodenectomy

Overall, available evidence suggests that Braun enteroenterostomy may reduce the incidence or severity of DGE in select settings; however, findings remain inconsistent, and the lack of high-quality prospective data limits definitive conclusions (Figure 4).

Beyond reconstructive configuration, the extent of gastric and pyloric preservation has also been proposed as a determinant of DGE.

Classic PD vs. PPPD

**Figure 5:**
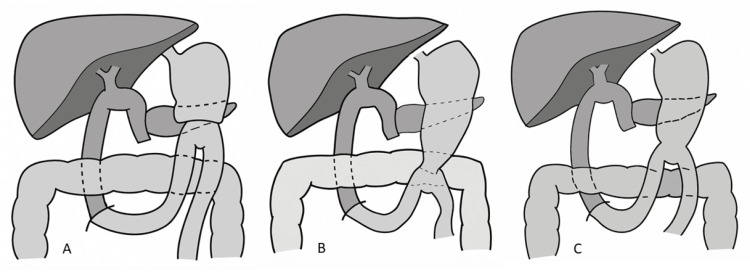
Schematic comparison of BII antecolic reconstruction according to the type of pancreatoduodenectomy (A) Classical PD (Whipple): BII antecolic gastrojejunostomy following standard resection including antrectomy. (B) PPPD: BII antecolic duodenojejunostomy with preservation of the pylorus. (C) PrPD: BII antecolic gastrojejunostomy performed after pyloric resection. Clinical relevance: The extent of gastric and pyloric preservation influences postoperative gastric motility and may affect the risk and pattern of DGE across classical, PPPD, and PrPD. This schematic illustration is not drawn to anatomical scale and is intended solely to represent the reconstruction pathway. BII, Billroth II; PD, pancreaticoduodenectomy; PPPD, pylorus-preserving PD; PrPD, pylorus-resecting pancreaticoduodenectomy

Retrospective, prospective, and randomized studies have consistently shown no significant difference in the incidence of DGE between classic PD and PPPD [20, 39-42]. In a recent retrospective single-center study, Gamboa-Hoil et al. [43] reported lower DGE rates among PPPD patients undergoing retrocolic BII reconstruction; however, outcomes in other cohorts have been variable. Current evidence indicates that the choice between classic PD and PPPD does not meaningfully influence the risk of DGE (Figure 5A, 5B).

Given the mixed results comparing classic PD and PPPD, further attention has focused on whether partial pyloric resection may influence postoperative motility.

Pylorus-Resecting PD (PrPPD) vs. PPPD

Several prospective randomized studies comparing PrPPD and PPPD have reported conflicting results. Kawai et al. [44] documented a lower incidence of DGE in the PrPPD group (4.5% vs. 17.2%; p = 0.0244), whereas Hackert et al. [45] and Matsumoto et al. [46] did not observe similar findings. In a meta-analysis limited to RCTs, Klaiber et al. [47] found no significant difference in DGE incidence between PrPPD and PPPD.

Although individual studies suggest a potential benefit of pylorus resection, current evidence does not demonstrate a consistent advantage over PPPD regarding the risk of DGE [48] (Figure 5C).

The contemporary literature has increasingly focused on preventing postoperative complications after PD, particularly evaluating the impact of specific reconstruction techniques [49]. Although the reconstruction technique may influence the development of DGE, findings remain heterogeneous across retrospective studies, prospective studies, randomized trials, and meta-analyses. Antimesenteric jejunal loop ascent, antecolic reconstruction, BII reconstruction, and Braun enteroenterostomy may reduce DGE in select contexts; however, no single approach has demonstrated consistent superiority across studies [5,48,50].

Table 1 and Figures 1-5 summarize and illustrate the reconstruction techniques discussed in this review. All images were created by the author, and although minor technical variations exist across studies, they represent the standard anatomical configuration for each procedure.

**Table 1:** Summary of studies evaluating DGE according to reconstruction technique after PD ^*^ Levels of evidence were assigned according to the Oxford Centre for Evidence-Based Medicine and correspond directly to the reported study design (e.g., RCT = Level 1b; prospective non-randomized study = Level 2b; retrospective cohort = Level 3b). ^**^ Stapled end-to-side duodenojejunostomy with alignment of gastric contours to facilitate gastric emptying. ^***^ Braun enteroenterostomy performed proximal to the gastrojejunal anastomosis. AC, antecolic; AM, antemesenteric; BEE, Braun enteroenterostomy; BI, Billroth I; BII, Billroth II; BPL, biliopancreatic limb; CPD, classic pancreaticoduodenectomy; DGE, delayed gastric emptying; NR, not reported; PD, pancreaticoduodenectomy; PrPD, pylorus-resecting pancreaticoduodenectomy; PPPD, pylorus-preserving pancreaticoduodenectomy; RC, retrocolic; RM, retromesenteric; RCT, randomized controlled trial; R-Y, Roux-en-Y reconstruction; SSPPD, subtotal stomach-preserving pancreaticoduodenectomy

Author, year, and country	Study design (period)	Oxford level^*^	Surgeons	Procedure	BPL route	Jejunal loop position	Reconstruction type	Histology	Incidence of DGE	Outcome measure	p-Value
Antimesenteric vs. retromesenteric jejunal loop ascent											
Park et al. (2003) [11], Korea	Retrospective (1996-2000)	3b	Single surgeon	PPPD (2-4 cm duodenum preserved)	Retromesenteric (n = 104) / Antimesenteric (n = 46)	Left	Antecolic	Benign/Malignant	24%	RM: 31.7% / AM: 6.5%	<0.05
Butler et al. (2004) [12], Canada	Retrospective (1998-2000)	3b	Senior residents under the supervision of two experts	PrPD / PPPD	Retromesenteric (n = 38) / Antimesenteric (n = 40)	Left	Retromesenteric / Antimesenteric	Benign/Malignant	28%	RM: mean 11 / AM: mean 7.2 days	<0.05
Antecolic vs. retrocolic gastroenteric reconstruction											
Tani et al. (2006) [13], Japan	RCT (2002-2004)	1b	NR	PPPD (3 cm duodenum preserved)	Antimesenteric	Right	Antecolic (n = 20) / Retrocolic (n = 20)	Benign/Malignant	27.50%	AC: 5% / RC: 50%	0.0014
Tamandl et al. (2014) [14], Austria	RCT (2007-2009)	1b	NR	PPPD	Antimesenteric	Right	Antecolic (n = 36) / Retrocolic (n = 28)	Benign/Malignant	20%	AC: 17.6% / RC: 23.1%	0.628
Kakaei et al. (2019) [15], Iran	RCT (2016-2017)	1b	Surgical staff	PPPD (2 cm duodenum preserved)	Antimesenteric	NR	Antecolic (n = 15) / Retrocolic (n = 15)	Periampullary tumor	16.60%	AC: 13% / RC: 20%	0.75
Gangavatiker et al. (2011) [16], India	RCT (2006-2008)	1b	NR	CPD / PPPD (3 cm duodenum preserved)	Antimesenteric	Right	Antecolic (n = 32) / Retrocolic (n = 36)	Benign/Malignant	30.90%	AC: 34.4% / RC: 27.8%	0.6
Eshuis et al. (2014) [17], Netherlands	RCT (2009-2011)	1b	10 hospitals	PPPD (2-4 cm duodenum preserved) / CPD	Antimesenteric	Left	Antecolic (n = 121) / Retrocolic (n = 125)	Benign/Malignant	61%	AC: 61% / RC: 60%	0.89
Sahora et al. (2015) [18], USA	Retrospective (2000-2012)	3b	One surgeon (antecolic) vs. three surgeons (retrocolic)	PD with antrectomy	Antimesenteric	NR	Antecolic (n = 400) / Retrocolic (n = 400)	Benign/Malignant	18%	AC: 15% / RC: 21%	0.021
BI vs. BII reconstruction											
Goei et al. (2001) [21], Netherlands	Retrospective (Amsterdam: 1992-1996; Groningen: 1988-1998)	3b	NR	PPPD Amsterdam: BII / Groningen: BI	Antimesenteric	NR	BII (n = 123) / BI (n = 51)	Benign/Malignant	44.80%	BII: 32% / BI: 76%	<0.05
Ohwada et al. (2002) [23], Japan	Prospective non-randomized (1997-2000)	2b	NR	PPPD / CPD	NR	NR	BI PPPD (n = 28) / CPD (n = 26)	Benign/Malignant	16%	PPPD: 36% / CPD: 4%	NR
Ueno et al. (2009) [24], Japan^**^	Prospective (2005-2007)	2b	NR	PPPD (4-6 cm duodenum preserved)	Antimesenteric	Right	BI (n = 12)	Malignant	0%	0%	-
BII vs. Roux-en-Y reconstruction											
Shimoda et al. (2013) [25], Japan	RCT (2008-2011)	1b	NR	SSPPD	Antimesenteric	NR	Antecolic BII (n = 52) / R-Y (n = 49)	Benign/Malignant	12.80%	BII: 5.7% / R-Y: 20.4%	0.028
Busquets et al. (2019) [26], Spain	RCT (2013-2015)	1b	Single center	NR	NR	NR	Antecolic BII (NR) / R-Y (NR)	Malignant	45%	BII: 45% / R-Y: 45%	1
Herrera-Cabezón et al. (2018) [27], Spain	RCT (2013-2016)	1b	Single center	CPD	NR	NR	BII (n = 32) / R-Y (n = 32)	Benign/Malignant	20.30%	BII: 25% / R-Y: 15.6%	0.35
Ke et al. (2013) [28], China	RCT (2006-2012)	1b	Multicenter single surgeon	CPD	Antimesenteric	Right	Antecolic BII (n = 109) / Isolated R-Y (n = 107)	Benign/Malignant	-	BII: 25% / Isolated R-Y: 23%	>0.05
Tani et al. (2014) [29], Japan	RCT (2009-2012)	1b	NR	PrPD / CPD	Antimesenteric	Right	BII (n = 76) / Isolated R-Y (n = 75)	Benign/Malignant	13.20%	BII: 12% / Isolated R-Y: 15%	0.609
Braun enteroenterostomy vs. no Braun enteroenterostomy											
Nikfarjam et al. (2012) [31], Australia	Retrospective (2009-2011)	3b	Single surgeon	CPD	Antimesenteric	NR	Antecolic/BII BEE (n = 24) / No BEE (n = 20)	Benign/Malignant	18.20%	BEE: 4.2% / No BEE: 35%	0.008
Zhang et al. (2014) [32], China	Retrospective (2009-2013)	3b	6 surgeons	CPD	Antimesenteric	NR	Antecolic or Retrocolic BII BEE (n = 347) / No BEE (n = 48)	Benign/Malignant	11.30%	BEE: 10.7% / No BEE: 16.7%	0.22
Xu et al. (2014) [33], China	Retrospective (2000-2013)	3b	NR	CPD	Antimesenteric	NR	Antecolic/BII BEE (n = 206) / No BEE (n = 201)	Benign/Malignant	16.70%	BEE: 6.7% / No BEE: 26.9%	0.001
Wang et al. (2014) [34], China^***^	Prospective (2008-2012)	2b	NR	CPD	Antimesenteric	NR	Antecolic/BII BEE (n = 32) / No BEE (n = 30)	Benign/Malignant	24.10%	BEE: 28% / No BEE: 20%	0.455
Watanabe et al. (2015) [35], Japan	Retrospective (2008-2013)	3b	6 surgeons / 9 residents	PPPD (3-5 cm duodenum preserved)	Antimesenteric	NR	Antecolic/BII BEE (n = 98) / No BEE (n = 87)	Benign/Malignant	11.80%	BEE: 4% / No BEE: 21%	0.01
Imamura et al. (2014) [36], Japan	RCT (2005-2011)	1b	Same surgical team	PPPD (2.4 cm duodenum preserved)	Antimesenteric	Right	BII BEE Antecolic (n = 58) / Retrocolic vertical (n = 58)	Benign/Malignant	16.40%	AC: 12.1% / RC: 20.7%	0.31
Cordesmeyer et al. (2014) [37], Germany	Retrospective (2004-2011)	3b	NR	PPPD	Antimesenteric	Right	No BEE / retrocolic short loop (n = 40), No BEE / retrocolic long loop (n = 22), BEE / retrocolic long loop (n = 23), BEE / antecolic long loop (n = 28)	Periampullary and pancreatic head tumor	-	No BEE / retrocolic short: 46.8%, No BEE / retrocolic long: 25%, BEE / retrocolic long: 28.6%, BEE / antecolic long: 4.5%	0.009
Classic PD vs. PPPD											
Di Carlo et al. (1999) [39], Italy	Retrospective (1990-1997)	3b	NR	CPD / PPPD (2 cm duodenum preserved)	NR	NR	CPD (n = 39) / PPPD (n = 74)	Malignant	13.20%	CPD: 15.3% / PPPD: 12.1%	>0.05
Horstmann et al. (2004) [40], German	Prospective non-randomized (1994-2000)	2b	NR	CPD / PPPD (2 cm duodenum preserved)	NR	NR	Antecolic CPD (n = 19) / PPPD (n = 113)	Malignant	12.80%	CPD: 21% / PPPD: 12%	0.2688
Tran et al. (2004) [41], Netherlands	RCT (1992-2000)	1b	Multicenter	CPD / PPPD (2 cm duodenum preserved)	NR	NR	CPD (n = 83) / PPPD (n = 87)	Benign/Malignant	21.70%	CPD: 23% / PPPD: 22%	0.8
Seiler et al. (2005) [42], Switzerland	RCT (1996-2001)	1b	NR	CPD / PPPD	NR	NR	CPD (n = 66) / PPPD (n = 64)	Benign/Malignant	38.50%	CPD: 45% / PPPD: 31%	0.096
Gamboa-Hoil et al. (2025) [43], Mexico	Retrospective (2021-2022)	3b	Single surgeon	CPD / PPPD (2 cm duodenum preserved)	Retrocolic	Left	CPD (n = 9) / PPPD (n = 6)	Malignant	46%	CPD: 77% / PPPD: 0%	0.007
PrPPD vs. PPPD											
Kawai et al. (2011) [44], Japan	RCT (2005-2009)	1b	Single center	PrPD (Adjacent to pylorus) / PPPD (3-4 cm duodenum preserved)	NR	NR	PrPD (n = 66) / PPPD (n = 64)	Periampullary / pancreatic tumor	10.70%	PrPD: 4.5% / PPPD: 17.2%	0.0244
Hackert et al. (2018) [45], Germany	Blinded RCT (2013-2016)	1b	Single center	PrPD (1 cm proximal duodenum resected) / PPPD (2 cm duodenum preserved)	Antimesenteric	Right	Antecolic PrPD (n = 93) / PPPD (n = 95)	Benign/Malignant	28.10%	PrPD: 31.2% / PPPD: 25.3%	0.367
Matsumoto et al. (2014) [46], Japan	RCT (2003-2009)	1b	1 of 2 surgeons	PrPD (3 cm proximal duodenum resected) / PPPD (3 cm duodenum preserved)	NR	NR	Retrocolic PrPD (n = 50) / PPPD (n = 50)	Benign/Malignant	16%	PrPD: 12% / PPPD: 20%	0.414

Limitations

Taken together, the variability of results across studies highlights inherent limitations in the current evidence, underscoring the need for a dedicated appraisal of methodological constraints. A formal meta-analysis was not performed due to substantial heterogeneity among studies in design, patient populations, reconstruction techniques, and the inconsistent reporting of effect sizes and confidence intervals, which precluded meaningful statistical pooling.

Additionally, the predominance of retrospective cohorts, single-center experiences, and surgeon-dependent technical variations introduces potential selection and reporting biases. Further variability in sample size, perioperative protocols, follow-up duration, and documentation of confounding factors, such as postoperative complications, also limits comparability across studies. Finally, because this review is narrative rather than systematic, publication bias and incomplete reporting cannot be excluded.

## Conclusions

Current evidence indicates that no single reconstructive approach has demonstrated consistent superiority. Variability in surgical expertise, anatomical configuration, and perioperative management likely contributes to these inconsistent findings. Well-designed, prospective, multicenter studies are needed to clarify the true impact of reconstruction technique on postoperative gastric function and to guide standardized, evidence-based surgical decision-making.
